# Corneal biomechanical parameters in keratoconus eyes with abnormal elevation on the back corneal surface only versus both back and front surfaces

**DOI:** 10.1038/s41598-021-91263-7

**Published:** 2021-06-07

**Authors:** Mohammad-Reza Sedaghat, Hamed Momeni-Moghaddam, Cynthia J. Roberts, Nasim Maddah, Renato Ambrósio, Seyed Rafi Hosseini

**Affiliations:** 1 Eye Research Center, Mashhad University of Medical Sciences, Mashhad, Iran; 2 Health Promotion Research Center, Zahedan University of Medical Sciences, Zahedan, Iran; 3 Ophthalmology and Visual Sciences, Biomedical Engineering, The Ohio State University, Columbus, OH 43210 USA; 4 Department of Optometry, School of Paramedical Sciences, Mashhad University of Medical Sciences, Mashhad, Iran; 5 Rio de Janeiro Corneal Tomography and Biomechanics Study Group, Rio de Janeiro, Brazil; 6 Department of Ophthalmology, Federal University of São Paulo, São Paulo, Brazil; 7 Department of Ophthalmology, Federal University of the State of Rio de Janeiro, Rio de Janeiro, Brazil; 8 Department of Optometry, School of Rehabilitation Sciences, Zahedan University of Medical Sciences, Zahedan, Iran

**Keywords:** Corneal diseases, Medical research, Eye manifestations

## Abstract

Corneal biomechanical parameters were compared in 100 keratoconus eyes with abnormal elevation on the back corneal surface only (group 1), versus both the back and front surfaces (group 2). Scheimpflug tomography with Pentacam HR, corneal biomechanical assessments using Corvis ST and Ocular Response Analyzer (ORA) and corneal epithelium thickness maps using anterior segment optical coherence tomography were assessed. There were no significant differences in the IOP measured using Corvis ST and ORA, age or sex between the two groups. Statistically significant differences were found in all corneal shape parameters and all new parameters of Corvis ST: corneal stiffness parameter at first applanation (SP-A1), integrated inverse radius (IR) and deformation amplitude ratio (DAR)) between groups (p < 0.001). The classic parameters of ORA including corneal hysteresis (CH) and corneal resistance factor (CRF) were about 1.00 mmHg higher in group 1 (p < 0.001). In conclusion, keratoconus eyes with abnormal elevation limited to the back corneal surface have lower grade, stiffer corneal biomechanical parameters and less asymmetric shape. This is consistent with progressive biomechanical weakening from the first detectable back surface elevation to manifestation on the front surface as the severity overwhelms the ability of the epithelium to compensate.

## Introduction

Keratoconus is a non-inflammatory progressive corneal ectatic disease associated with corneal thinning and abnormal bulging at one or both corneal surfaces, which leads to irregular astigmatism and visual impairment^1^. Although readily diagnosed based on the slit-lamp’s findings in the advanced stages, detection of keratoconus in the very early stages can be challenging^2^. Although corneal topography is a sensitive method to detect keratoconus before presentation of slit-lamp findings, it assesses only the anterior corneal surface, while the tomographic techniques perform a complete corneal architecture analysis to provide important data, such as posterior corneal surface analysis and pachymetric profile^3^.

Presence of abnormal elevation on the back corneal surface was reported as a primary sign of early ectatic change in keratoconus and may be used in the differential diagnosis of keratoconic corneas from normal corneas; however, its effectiveness in detecting subclinical forms of keratoconus has not been confirmed^4^. Therefore, the abnormal elevation values limited to the back corneal surface cannot be used alone for detecting or staging/classification of keratoconus.

With the popularity of corneal excimer laser refractive surgery techniques, the diagnosis of keratoconus in very early stages is vital in preventing iatrogenic ectasia. Recent evidence has shown the complementary role of corneal biomechanical features in discriminating keratoconic corneas from the normal ones^5–7^, and its role in detecting keratoconus in the initial stages prior to the topographical manifestations of keratoconus^8^.

The availability of instruments to access in vivo corneal biomechanical parameters, as the most probable primary sign of keratoconus, has greatly increased the diagnostic accuracy of keratoconus^9,10^. Although ex-vivo evaluations relying on the stress–strain assessments of the cornea provide an estimation of classic mechanical properties such as elastic modulus; this is not appropriate for clinical use which must rely on a nondestructive load to assess biomechanical response. Two commercial instruments available for clinical evaluation of corneal biomechanics are the Corvis ST (Oculus; Wetzlar, Germany) and Ocular Response Analyzer (ORA, Reichert Ophthalmic Instruments, Buffalo, NY, USA). Among the newer parameters of the Corvis ST, the stiffness parameter at the first applanation (SP-A1) was reported as a novel stiffness parameter reflecting the overall resistance of the cornea to deformation^11^.

In some keratoconus cases, the subtraction elevation values compared to the reference surface on the front corneal surface are within normal limits while the back values ​​are abnormal. Our literature review showed no data available on the comparison of biomechanical parameters of the cornea in keratoconic patients with abnormal elevation values on both corneal surfaces vs those with abnormal values only on the posterior surface. Therefore, the purpose of the current study was to compare the corneal biomechanical response parameters in these two groups of keratoconic eyes.

## Methods

One hundred keratoconus eyes of 91 patients who had abnormal elevation values on only the posterior corneal surface (group 1, n = 47 eyes) or both anterior and posterior corneal surfaces (group 2, n = 53) were included in this cross-sectional study. Keratoconus was diagnosed based on slit-lamp findings (e.g. Fleischer ring, stress line) or scissoring reflex on retinoscopy and confirmed by an expert corneal specialist. It should be emphasized that not all signs are present in all cases, for example, the Vogt striae sign is less common in cases where only posterior elevation is abnormal, while in almost all cases retinoscopic reflex was irregular. Tenets of the Declaration of Helsinki were respected in all steps of the current study and informed consent was obtained from all patients after explaining the objectives of this study. The study protocol was approved by the Ethics Committee of Mashhad University of Medical Sciences. (Code: 961181).

Participants with history of keratorefractive surgery, ocular surgery, corneal cross-linking or corneal rings implantation; severe dry eye, pregnancy at the time of examination, history of corneal scar or hydrops and other eye diseases except keratoconus; previous eye trauma; collagen-vascular disorders and diabetic mellitus were excluded from the study.

Standard ophthalmic examination included distance corrected and uncorrected visual acuity recorded in decimal notation (DUVA & DCVA) and refractive status (sphere, spherical equivalent (SE), magnitude and axis of total astigmatism).

Simulated mean keratometry (Km: average of the flat and steep meridians), magnitude and axis of corneal astigmatism (CA) were measured using TMS-4 (Tomey Corp, Nagoya, Japan).

Corneal tomography based on Scheimplfug technique was done using Pentacam HR (Oculus; Wetzlar, Germany). Extracted parameters included the front and back surfaces’ mean keratometry; corneal astigmatisms (CA), and central corneal thickness at the apex (CCT) and the thinnest point (CTP), thickness progression index along the all corneal meridians starting from CTP (average (PPIave) and maximum (PPImax) pachymetric progression index) , maximum Ambrosio relational thickness (ART = CTP/PPImax), ART along the horizontal (temporal-nasal) meridian (ARTh), inferior-superior asymmetry value (IS) and Belin-Ambrosio enhanced ectasia total deviation index (BAD-D) which is a combination of the five deviation indices using a linear regression analysis.

In-vivo corneal biomechanical assessments were done using corneal visualization Scheimpflug technology (Corvis ST, Oculus; Wetzlar, Germany) and Ocular Response Analyzer based on bidirectional applanation tonometry technique (ORA, Reichert Ophthalmic Instruments, Buffalo, NY, USA).

Extracted dynamic corneal response parameters using Corvis ST were those provided in the ARV (Ambrosio, Roberts, Vinciguerra) printout including stiffness parameter at first applanation (SP-A1, resultant pressure [adjusted pressure at AI (adj AP1)—biomechanically compensated IOP (bIOP)] divided by deflection amplitude at A1)^11^, integrated radius (IR, area under the inverse concave radius curve), deformation amplitude ratio (DAR, the ratio between DA at the apex and the average of DA at 2 mm around the center in temporal and nasal directions)^12^.

Classic pressure-derived parameters from the ORA were corneal hysteresis (CH), corneal resistance factor (CRF), two IOP parameters. (IOPcc: corneal compensated IOP, IOPg: Goldmann correlated IOP).

Pachymetry and epithelium thickness at the center and the thinnest point were measured using anterior segment optical coherence tomography (AS-OCT, Optovue, Inc., CA, USA).

Patients wearing contact lenses were requested to stop wearing them at least 2 weeks for soft and 4 weeks for rigid contact lenses before corneal assessments.

To divide the studied eyes into two groups based on abnormal elevation limited to the back corneal surface (group 1) or abnormal values at both corneal surfaces (group 2), the reference surface used in Pentacam for elevation mapping was the 8 mm best fit sphere (BFS) on the front and back corneal surfaces. Abnormal elevation was defined based on values greater than 12 μm on the front elevation map and 15 μm on the back elevation map^13,14^.

Data were analyzed in SPSS.22 software. The normality of the data were assessed using the Kolmogorov–Smirnov test. The mean of different variables between the two groups were compared using the independent samples-T test for parameters with normal distribution and the Mann–Whiney U test for parameters with non-normal distribution. The Chi-square test was used to compare different grades of keratoconus between the two groups. The significance level was considered as p < 0.05 in all tests.

### Ethical approval

The study protocol was approved by the local ethics committee. Subjects’ informed consent was obtained following the Principles of Declaration of Helsinki.

## Results

The mean age in all patients was 23.7 ± 6.2 years and in the group with abnormal elevation limited to the back corneal surface and both corneal surfaces were 23.9 ± 5.2 and 23.6 ± 7.0 years, respectively (p = 0.452).

The percentage of females and males in the group with abnormal elevation limited to the back surface was 40.4% and 59.6%, respectively and in the other group 35.8% and 64.2%, respectively (p = 0.638).

Mean and SD of IOPnct, IOPg and IOPcc was 14.42 ± 1.15, 11.87 ± 2.84 and 13.89 ± 2.74 mmHg in the group with abnormal elevation limited to the back surface and 14.15 ± 1.00, 10.97 ± 2.17 and 14.03 ± 2.12 mmHg in the other group, respectively. The two groups were not significantly different based on the IOP measured using Corvis ST [IOPnct (p = 0.332)) and ORA (IOPg (p = 0.079) and IOPcc (p = 0.447)].

Mean values and standard deviations of visual acuity, refraction and topography data are presented in Table 1.

**Table 1:** Mean and SD of visual acuity, refractive errors and topography data separately in the two groups (n = 100 eyes).

Abnormal elevation	Back corneal surface (n = 47)		Both corneal surfaces (n = 53)		p value
	Mean ± SD (95% CI)	Range	Mean ± SD (95% CI)	Range	
**Variables**					
UDVA (decimal)	0.51 ± 0.29 (0.42, 0.60)	0.08 to 1.00	0.32 ± 0.22 (0.26, 0.38)	0.01 to 0.90	0.002
CDVA (decimal)	0.93 ± 0.11 (0.89, 0.96)	0.60 to 1.00	0.70 ± 0.23 (0.64, 0.77)	0.08 to 1.00	< 0.001
Sphere (D)	− 0.89 ± 1.19 (− 1.24, -0.54)	− 3.75 to 1.00	− 2.03 ± 2.81 (− 2.81, − 1.26)	− 11.50 to 2.75	0.071
Cylinder (D)	− 1.73 ± 1.17 (− 2.08, − 1.39)	− 4.75 to 0.00	− 4.14 ± 1.90 (− 4.66, − 3.61)	− 8.50 to − 0.25	< 0.001
Axis (º)	87.11 ± 63.48 (68.47, 105.75)	0.0 to 180	90.42 ± 59.47 (74.02, 106.81)	3.0 to 179.0	0.920
SE (D)	− 1.76 ± 1.34 (− 2.15, − 1.36)	− 5.00 to 0.38	− 4.10 ± 3.10 (− 4.96, − 3.25)	− 14.38 to − 0.13	< 0.001
Mean-KR (D)	45.24 ± 1.87 (44.69, 45.79)	41.09 to 49.42	48.89 ± 3.41 (47.95, 49.83)	42.89 to 60.11	< 0.001
CA (D)	2.40 ± 1.43 (1.98, 2.82)	0.35 to 5.88	5.25 ± 1.90 (4.72, 5.77)	1.41 to 9.31	< 0.001*
CA-Axis (º)	98.82 ± 63.41 (80.21, 117.44)	3.00 to 177.00	86.30 ± 58.13 (70.27, 102.32)	1.00 to176.00	0.337

*SD* standard deviation, *CI* confidence interval, *UDVA* uncorrected distance visual acuity, *CDVA* corrected distance visual acuity, *SE* spherical equivalent, *KR* keratometry reading, *CA* corneal astigmatism; *parametric statistics.

Statistical analysis using the Mann–Whitney U test showed significant differences in all parameters except the spherical component of refractive errors and the axes of refractive and corneal astigmatisms. A deterioration of at least two lines of visual acuity was observed in the group with abnormal elevation at both corneal surfaces which was accompanied by up 2.0–2.5 D more negative results in comparing the spherical equivalent and refractive cylinder between the two groups. The mean keratometry was 3.65 D flatter in the group with abnormal elevation limited to the back corneal surface.

The mean and standard deviations of some Pentacam indices are shown in Table 2.

**Table 2:** Mean and SD of corneal curvature, best fit sphere radius, maximum elevation, thickness and thickness profile using Pentacam separately in the two groups (n = 100 eyes).

Abnormal elevation	Back corneal surface (n = 47)		Both corneal surfaces (n = 53)		P value
	Mean ± SD (95% CI)	Range	Mean ± SD (95% CI)	Range	
**Variables**					
Front mean-KR (D)	44.97 ± 1.77 (44.45, 45.49)	41.20 to 48.70	48.46 ± 3.18 (47.58, 49.34)	42.55 to 59.50	< 0.001
Front CA (D)	2.08 ± 1.43 (1.66, 2.50)	− 3.30 to 5.00	3.79 ± 1.56 (3.36, 4.22)	− 0.10 to 6.60	< 0.001*
Front CA-axis (º)	97.90 ± 51.91 (82.66, 113.14)	0.30 to 179.80	85.44 ± 50.87 (71.42, 99.47)	2.20 to 168.10	0.191
Front BFS radius (mm)	7.70 ± 0.29 (7.62, 7.79)	7.12 to 8.53	7.36 ± 0.34 (7.27, 7.46)	6.15 to 8.33	< 0.001*
Front maximum elevation (µm)	11.04 ± 2.68 (10.24, 11.85)	6 to 16	29.08 ± 9.05 (26.51, 31.65)	18.0 to 60.0	< 0.001
Front K-max (D)	48.28 ± 2.29 (47.61, 48.96)	44.70 to 54.30	55.92 ± 4.99 (54.54, 57.30)	46.20 to 70.40	< 0.001
I-S asymmetry (D)	2.16 ± 1.28 (1.78, 2.54)	− 1.50 to 4.50	6.57 ± 2.58 (5.86, 7.29)	2.30 to 14.30	< 0.001
Back mean-KR (D)	− 6.52 ± 0.34 (− 6.62, − 6.42)	− 7.25 to − 5.75	− 7.17 ± 0.65 (− 7.35, − 6.99)	− 9.70 to − 6.25	< 0.001
Back CA (D)	− 0.54 ± 0.22 (− 0.60, − 0.47)	− 1.10 to − 0.10	− 0.81 ± 0.26 (− 0.88, − 0.74)	− 1.40 to − 0.30	< 0.001
Back CA-axis (º)	104.52 ± 48.08 (90.41, 118.64)	16.50 to 179.50	91.40 ± 51.68 (77.15, 105.64)	1.70 to 173.90	0.216
Back BFS radius (mm)	6.31 ± 0.37 (6.20, 6.42)	4.45 to 7.14	6.16 ± 0.26 (6.09, 6.24)	5.68 to 6.82	0.029*
Back maximum elevation (µm)	30.27 ± 7.48 (28.02, 32.51)	18 to 44	57.92 ± 12.85 (54.27, 61.57)	37.0 to 88.0	0.001*
CCT (µm)	494.76 ± 28.09 (486.51, 503.01)	433 to 558	458.52 ± 29.47 (450.40, 466.65)	388 to 533	< 0.001*
CTP (µm)	482.85 ± 27.60 (474.75, 490.95)	426 to 545	447.64 ± 29.07 (439.62, 455.65)	377 to 515	< 0.001*
ART (µm)	219.51 ± 44.77 (206.36, 232.65)	147.00 to 317.00	152.83 ± 43.92 (140.72, 164.93)	35.00 to 313.00	< 0.001*
ARTh (µm)	299.58 ± 63.28 (280.99, 318.16)	197.90 to 479.50	181.71 ± 66.20 (163.46, 199.96)	50.80 to 430.80	< 0.001*
PImax	2.27 ± 0.38 (2.16, 2.38)	1.62 to 3.15	3.15 ± 1.09 (2.85, 3.45)	1.51 to 9.28	< 0.001
PIave	1.54 ± 0.21 (1.48, 1.61)	1.22 to 2.03	2.12 ± 0.71 (1.93, 2.32)	1.01 to 6.36	< 0.001
BAD-D	4.70 ± 1.60 (4.23, 5.18)	2.75 to 12.47	9.01 ± 3.25 (8.12, 9.91)	3.35 to 19.87	< 0.001

*SD* standard deviation, *CI* confidence interval, *KR* keratometry reading, *CA* corneal astigmatism, *BFS* best fit sphere, *CCT* central corneal thickness, *CTP* corneal thinnest point, *ART* ambrosio relational thickness, *ARTh* ambrósio’s relational thickness to the horizontal profile, *PPImax* maximum pachymetric progression index, *PPIave* average pachymetric progression index, *IS* inferior-superior difference value, *BAD-D* Belin-Ambrosio Enhanced Ectasia Total Deviation Index; *parametric statistics.

Table 2 shows steeper keratometry readings in both corneal meridians in the front and back corneal surfaces in the group with abnormal elevations at both corneal surfaces with significant differences in all obtained parameters except the corneal astigmatism’s axes in both surfaces. The highest difference in the Keratometry reading (KR) was related to the front maximum keratometry with a mean difference 7.63 D; also the I–S asymmetry value had a mean difference 4.41 D between the two groups.

The corneal thickness at the apex, the thinnest point and the progression indices were significantly different between the two groups. The mean differences in CCT and CTP in the two groups were 36.24 and 35.21 µm, respectively.

The BFS radius was significantly steeper in the group with abnormal elevations at both corneal surfaces than in the group with abnormal elevations limited to the posterior corneal surface (p < 0.05).

By examining the elevation data relative to the BFS, it is evident that there is significant difference in the maximum elevation values in the front (p < 0.001) and back (p = 0.001) corneal surfaces with greater elevation in relation to the BFS was observed in the group with abnormal elevations at both corneal surfaces.

**Figure 1:**
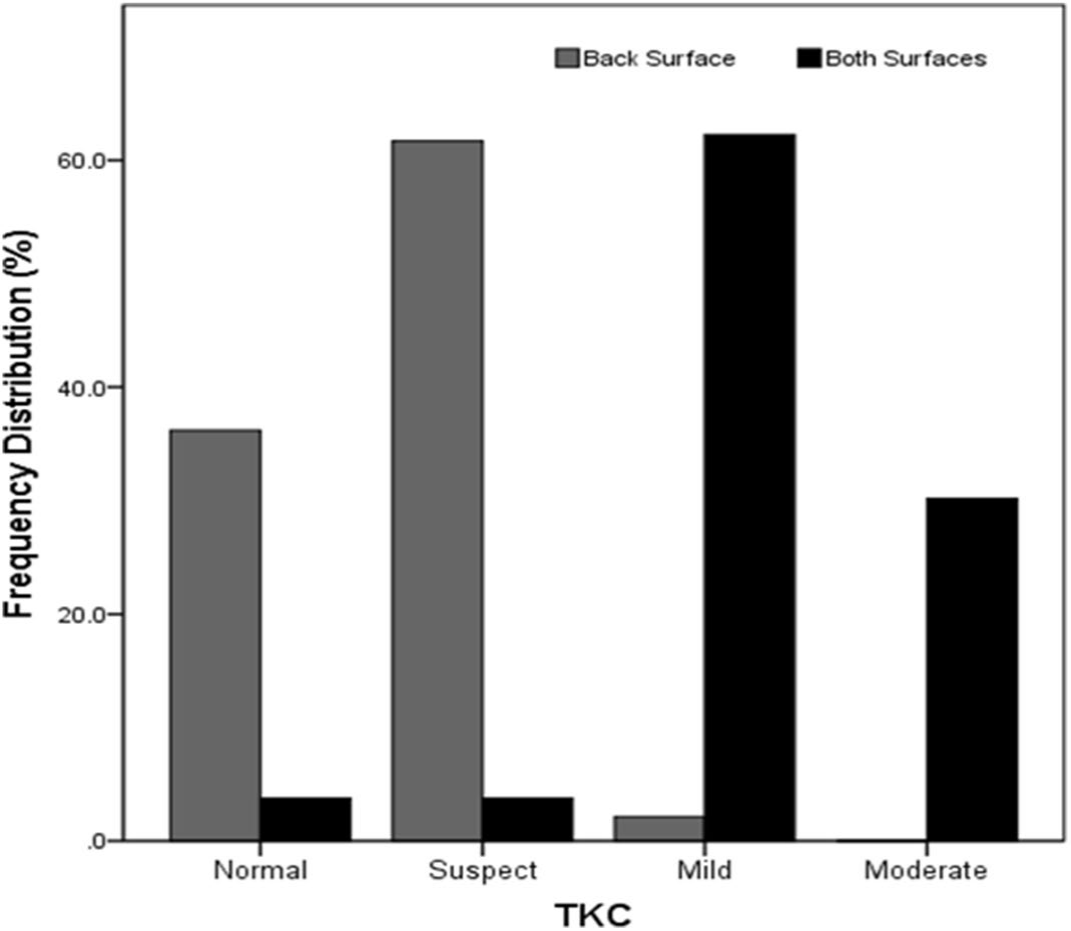
Frequency distribution of different grades based on the Topographic Keratoconus Classification (TKC) separately in the two groups (n = 100 eyes).

Keratoconus staging was done based on the topographic keratoconus classification (TKC) provided by Pentacam which includes five grades: 0 (normal), 1 (suspect), 2 (mild), 3 (moderate) or 4 (severe keratoconus). In some cases, the system displays the intermediate grades, for example 2–3, in these cases; the lower number was recorded for the analysis^15^ (Fig. 1).

It is evident that the group with abnormal elevation limited to the back corneal surface had the lower grades compared to the other group. The Chi square test showed a significant difference in the distribution of different grades of keratoconus in the two groups (X^2^ = 81.409, df = 3, p < 0.001).

The pachymetry values in the center of the cornea (p < 0.001) and the thinnest point (p < 0.001) using the AS-OCT were significantly different between the two groups. These values in the group with abnormal elevations at both corneal surfaces were 472.38 ± 32.93 µm (95% CI 463.02, 481.74) and 444.24 ± 33.80 µm (95% CI 434.63, 453.85), respectively and in the group with abnormal elevation limited to the back surface were 498.55 ± 31.10 µm (95% CI 489.09, 508.00) and 480.50 ± 30.83 µm (95% CI 471.13, 489.87), respectively.

The epithelial thickness at the center (p = 0.002) and the thinness point (p < 0.001) of the cornea using AS-OCT were significantly thinner in the group with abnormal elevations at both corneal surfaces. The mean and SD of central and thinnest epithelial thicknesses in the group with abnormal elevation limited to the back surface were 53.18 ± 3.03 µm (95% CI 52.26, 54.11) and 46.36 ± 3.96 µm (95% CI 45.16, 47.57), respectively. These values in the other group were 50.74 ± 4.13 µm (95% CI 49.56, 51.92) and 42.62 ± 3.33 µm (95% CI 41.67, 43.57), respectively.

Corneal biomechanics assessment using ORA showed a significant difference in the mean CH and CRF between the two groups using the independent samples T test (p < 0.001). The mean and SD of CH in the group with abnormal elevation limited to the back surface and in the group with abnormal elevations at both corneal surfaces was 9.45 ± 1.23 (95% CI 9.08, 9.81) and 8.56 ± 1.12 (95% CI 8.25, 8.87) and the mean CRF was 8.51 ± 1.37 (95% CI 8.11, 8.92) and 7.52 ± 1.19 (95% CI 7.19, 7.85), respectively.

The mean difference in CH and CRF was 0.88 and 0.99 mmHg, respectively, which were significantly different between the two groups.

The new Corvis ST’s parameters are shown in Table 3.

**Table 3:** Mean and SD of the new parameters using Corvis ST in the two groups (n = 100 eyes).

Abnormal elevation	Back corneal surface (n = 47)		Both corneal surfaces (n = 53)		p value
	Mean ± SD (95% CI)	Range	Mean ± SD (95% CI)	Range	
**Variables**					
SPA1 (mmHg/mm)	80.75 ± 16.66 (75.86, 85.64)	42.80 to 110.80	61.34 ± 17.66 (56.47, 66.20)	23.00 to 93.90	< 0.001*
IR (mm^−1^)	9.01 ± 1.17 (8.66, 9.36)	6.30 to 11.50	10.70 ± 1.49 (10.29, 11.11)	6.90 to 14.60	< 0.001*
DAR	4.75 ± 0.55 (4.59, 4.92)	3.80 to 6.40	5.46 ± 0.72 (5.25, 5.66)	3.40 to 7.10	< 0.001*

*SD* standard deviation, *CI* confidence interval, *SP-A1* stiffness parameter at first applanation, *IR* integrated radius, *DAR* deformation amplitude ratio; *parametric statistics.

All new parameters of Corvis ST were significantly different using the independent samples-T test or its nonparametric equivalent (p < 0.05). The mean difference in SP-A1, IR and DAR were 19.41 mmHg/mm, 1.69 mm^−1^ and 0.70, respectively.

## Discussion

This study showed that the corneas in the group with abnormal elevation limited to the back corneal surface were stiffer biomechanically based on the new Corvis ST parameters (SPA1, IR, DAR). Also, the classic parameters from ORA (CH, CRF) showed significant reduction in group 2 with abnormal elevation in both surfaces. In addition, the corneal shape characteristics derived from Pentacam (curvature and thickness) indicated the thinner and steeper corneas in the group with abnormal elevation in both corneal surfaces.

These findings show that the early signs of ectasia are mainly detectable on the back corneal surface before the changes appear on the front surface where the epithelium can smooth irregularities. These results confirm previous studies. Tomidokoro et al.^16^ reported the back surface changes in the early stage of the keratoconus when investigating the quantitative changes in corneal curvature of both surfaces. In assessment of different geometric characteristics of keratoconus using the Pentacam, Miháltz et al.^17^ found the back elevation as the most essential diagnostic criterion for the eyes with keratoconus. Rao et al.^18^ in assessing the elevation maps in the eyes with suspicious Placido-disk based topography found increased back corneal surface elevation as risk factor and an early sign of form fruste keratoconus.

The current study showed that 97.9% of cases with only abnormal back elevation had grades of 0 and 1 while this value for the other group was 7.6%. On the other hand, 2.1% of cases had grades 2 and 3 in the group with abnormal elevation in the back surface, while 95.5% of cases with these grades were in the group with abnormal elevations in both surfaces. Considering the higher percentages of cases with lower grades of keratoconus in the group with abnormal elevation in the back corneal surface, this may be another reason for the detection of ectasia on the back surface first^16,18^.

Abnormal elevation on the back surface of the cornea has been reported as an early sign or one of the primary detectable ectatic changes, which can be used in differential diagnosis of keratoconus corneas from normal. However, its effectiveness in detecting subclinical keratoconus is less and therefore, these findings cannot be used alone for diagnostic purposes^4,19,20^. The results of the current study are consistent with back corneal changes being associated with earlier keratoconus with stiffer corneas and less change in front surface curvature. Once the disease has advanced to the point where the anterior stromal changes have overwhelmed the ability of the epithelium to compensate, abnormal front elevation is the result. The epithelium is thinner when both surfaces show abnormal elevation than when only the back surface is involved. This is consistent with epithelial thinning as a response to increasing curvature of the anterior stroma as keratoconus progresses^21^. A similar epithelial response to the underlying stromal curvature has also been reported in myopic refractive surgery which results not only in a thicker central epithelium in the central flatter area, but also thinner mid-peripheral epithelium in the area of greater post-operative curvature^22,23^.

Previous studies showed that there is a significant negative correlation between increased back corneal elevation and residual stromal bed thickness especially after laser in situ keratomileusis (LASIK) due to a lamellar cut in the stronger part of the cornea biomechanically, combined with tissue subtraction. In some cases, these changes may produce bulging of the back corneal surface following LASIK and finally iatrogenic ectasia^24–26^. Progressive increases in back surface elevation after refractive surgery may be associated with corneal biomechanical decompensation.

It has been proposed that progression in keratoconus is the result of a biomechanical cycle of decompensation^27^. The cycle is initiated by a focal weakening, potentially due to eye rubbing, that redistributes the biomechanical stress, resulting in thinning, that also redistributes the stress, resulting in increased curvature which again redistributes the stress, and the cycle continues. Focal weakening in keratoconus has been reported, both ex-vivo^28^, and in clinical measurements^29^, using confocal Brillouin microscopy. The current study provides further evidence that earlier stages of keratoconus with abnormal elevation only on the back surface have stiffer corneal responses than more advanced stages with abnormal elevation on both surfaces, consistent with a cycle of progressive weakening. As the curvature increases, the epithelium thins in response as an attempt to maintain the surface shape, with greater thinning in more advanced stages.

One limitation of this study was not including the additional parameters extracted from signal analysis of ORA that describe the waveform of the ORA’s response curve.

In conclusion, the present study showed that changes in the corneal biomechanical status known as the major potential etiologic factor for keratoconus are less in cases with abnormal elevation limited to the back surface of the cornea than those with abnormal elevations on both surfaces. This indicates definitively that early keratoconus is detectable first on the back surface, but is masked by the epithelium on the anterior surface, and is consistent with progressive biomechanical weakening.

## Data Availability

The datasetanalyzed for the current study is not available.
